# Mapping freezing tolerance QTL in alfalfa: based on indoor phenotyping

**DOI:** 10.1186/s12870-021-03182-4

**Published:** 2021-09-06

**Authors:** Laxman Adhikari, Shiva O. Makaju, Orville M. Lindstrom, Ali M. Missaoui

**Affiliations:** 1 Institute of Plant Breeding, Genetics and Genomics, The University of Georgia, Athens, GA USA; 2 Department of Horticulture, The University of Georgia, Athens, GA USA; 3 Department of Crop and Soil Sciences, The University of Georgia, Athens, GA USA

**Keywords:** Alfalfa, GBS, SNP, *CBF*, Freezing tolerance, Winter survival, Germplasms, Cold tolerance

## Abstract

**Background:**

Winter freezing temperature impacts alfalfa (*Medicago sativa* L.) persistence and seasonal yield and can lead to the death of the plant. Understanding the genetic mechanisms of alfalfa freezing tolerance (FT) using high-throughput phenotyping and genotyping is crucial to select suitable germplasm and develop winter-hardy cultivars. Several clones of an alfalfa F_1_ mapping population (3010 x CW 1010) were tested for FT using a cold chamber. The population was genotyped with SNP markers identified using genotyping-by-sequencing (GBS) and the quantitative trait loci (QTL) associated with FT were mapped on the parent-specific linkage maps. The ultimate goal is to develop non-dormant and winter-hardy alfalfa cultivars that can produce extended growth in the areas where winters are often mild.

**Results:**

Alfalfa FT screening method optimized in this experiment comprises three major steps: clone preparation, acclimation, and freezing test. Twenty clones of each genotype were tested, where 10 samples were treated with freezing temperature, and 10 were used as controls. A moderate positive correlation (r ~ 0.36, *P* < 0.01) was observed between indoor FT and field-based winter hardiness (WH), suggesting that the indoor FT test is a useful indirect selection method for winter hardiness of alfalfa germplasm. We detected a total of 20 QTL associated with four traits; nine for visual rating-based FT, five for percentage survival (PS), four for treated to control regrowth ratio (RR), and two for treated to control biomass ratio (BR). Some QTL positions overlapped with WH QTL reported previously, suggesting a genetic relationship between FT and WH. Some favorable QTL from the winter-hardy parent (3010) were from the potential genic region for a cold tolerance gene *CBF*. The BLAST alignment of a *CBF* sequence of *M*. *truncatula*, a close relative of alfalfa, against the alfalfa reference showed that the gene’s ortholog resides around 75 Mb on chromosome 6.

**Conclusions:**

The indoor freezing tolerance selection method reported is useful for alfalfa breeders to accelerate breeding cycles through indirect selection. The QTL and associated markers add to the genomic resources for the research community and can be used in marker-assisted selection (MAS) for alfalfa cold tolerance improvement.

**Supplementary Information:**

The online version contains supplementary material available at 10.1186/s12870-021-03182-4.

## Background

Most of the U.S. regions experience freezing to extremely low temperatures in winter posing a serious threat to the survival of herbaceous forage species. Severe winter injury in alfalfa is frequent especially in the northern climate [1, 2]. Harsh winters affect alfalfa (*Medicago sativa* L.) growth leading to reduced biomass yield, low stand persistence, and eventually death (http://www.canr.msu.edu/uploads/resources/pdfs/e2310.pdf). Improvement of alfalfa for winter hardiness (WH) has traditionally been achieved via mass and recurrent selection of superior genotypes in field nurseries in cold regions [3]. However, selection for cold hardiness in nurseries has often low efficiency because of the unpredictable winter season and the requirement of data from multiple locations and years for efficient selection [3]. Recording several data for multiple seasons is costly and laborious [4]. The North American Alfalfa Improvement Conference (NAAIC) also recommends data collection for a minimum of two locations and two years for winter survival evaluation (https://www.naaic.org/stdtests/wintersurvivalnew.pdf). The NAAIC also recommends some guidelines for the collection and interpretation of alfalfa winter survival data as the winter survival ratings recorded too early can underestimate the WH of dormant genotypes. Also, alfalfa selection against winter damage based on conventional breeding is too slow as the trait is quantitative and has substantial interaction with the environment [5]. Therefore, the assessment of WH in the field is a relatively tedious and long process.

Alfalfa is an autotetraploid (2n = 4x = 32) and the most widely cultivated forage globally [6]. Freezing tolerance (FT) is an important factor for predicting alfalfa winter hardiness (WH) [7]. Therefore, the evaluation of plant tolerance to freezing temperatures in a simulated environment is deemed as an alternative to field phenotyping. The indoor freezing tolerance tests are commonly carried out using a temperature bath [8], as electrolytic leakage assessment [9], using chlorophyll fluorescence assays [10], and freezer chambers with programmed temperatures [3]. The better nursery performance of plants selected from the indoor freezing test has been described in alfalfa [3, 11] and other species. Adkins et al. (2002) evaluated the cold hardiness of ten species of the genus *Hydrangea* in the lab, where they observed the performances of *H*. *macrophylla* cultivars corresponding to their landscape performance [12]. A strong positive correlation between the freezing test and plant cold hardiness was reported for St. Augustinegrass [*Stenotaphrum secundatum* (Walt.) Kuntze] [13].

Low temperature induces several genes and associated signal transduction pathways to synthesize essential biomolecules (proteins, soluble sugars, osmoprotectants) for cold acclimation [14]. The *CBF* (C-repeat binding factor) genes are transcription factors that play a key role in cold acclimation regardless of the source of temperatures [15, 16]. Cold-sensitive plants often lack the associated genes, or the genes get inactivated due to mutations thereby dysregulating the formation of regulatory and signaling molecules for cold acclimation. Other factors such as water stress, pesticide application, fertilizer treatment, bacterial colonization, and planting date [8] also affect plant cold hardiness. However, low temperature is the major factor bringing cellular changes and winter injuries in sensitive plants. Therefore, dissecting the genomic features associated with alfalfa freezing tolerance is crucial. Furthermore, some alfalfa germplasm, generally the cold-tolerant types, undergo dormancy when the days shorten and temperature drops in fall season and this phenomenon is called fall dormancy (FD). Nevertheless, recent findings indicated that winter survival (WS), the tendency of plants to withstand winter cold without injuries, and FD are two distinct traits and can be genetically separated to improve germplasm for both traits simultaneously [17]. Incorporation of cold tolerance in non-dormant alfalfa germplasm through genetic manipulation could be ideal for developing alfalfa cultivars that have high biomass yield and winter survival.

QTL analysis for identifying effective loci and genomic regions associated with traits of interest has been a common approach in crop breeding and genomics studies for the last couple of decades [18]. Poudel et al. [19] conducted QTL analysis on freezing tolerance in pseudo-F_2_ switchgrass population based on indoor phenotyping and reported six significant QTL and potential candidate genes. Recently, a group of researchers reported multiple QTL for cold acclimation and freezing tolerance related traits such as surviving green tissue and regrowth in Zoysiagrasses (*Zoysia* spp.) [20]. QTL analysis also revealed the genetic basis for adaptive freezing tolerance in locally adapted *Arabidopsis thaliana* populations from Italy and Sweden [21]. The closest diploid relative of alfalfa, *Medicago truncatula* was also investigated to detect QTL associated with the freezing damage and related traits such as foliar electrolyte leakage, chlorophyll content index, and dry weights [22]. The authors reported QTL on different linkage groups (LG) of *M*. *truncatula*: LG1, LG4, and LG6, where the freezing tolerance related QTL in LG6 was the most effective QTL explaining up to 40 % variation. In alfalfa, information is limited regarding effective freezing tolerance QTL that could also be useful for overall winter survival improvement of germplasm.

Winter-hardy non-dormant alfalfa with wider adaptation can fill the forage gap through the extension of regrowth time in winter and early spring. Seasonal forage gaps exist because of partial to complete growth cessation of warm-season species when cool-season forages are not productive yet [17]. Winters in Georgia and the southeast USA are overall mild, but these locations often experience a few freezing days per season depending on the latitude and elevation. Therefore, non-dormant and winter-hardy alfalfa are desirable to the region. In a previous study, we reported QTL associated with alfalfa WH and fall dormancy in field-grown plants [17].

In this study, we investigated the association of freezing tolerance with genetic loci using indoor phenotyping in freeze chambers. Accordingly, the objectives of this experiment were (i) to optimize a protocol for alfalfa freezing tolerance test in a walk-in freezer (ii) to identify alfalfa QTL associated with freezing tolerance, and (iii) to select freeze-tolerant advanced alfalfa breeding lines for the development of winter-hardy cultivars.

## Results

### Testing freezing tolerance

**Fig. 1:**
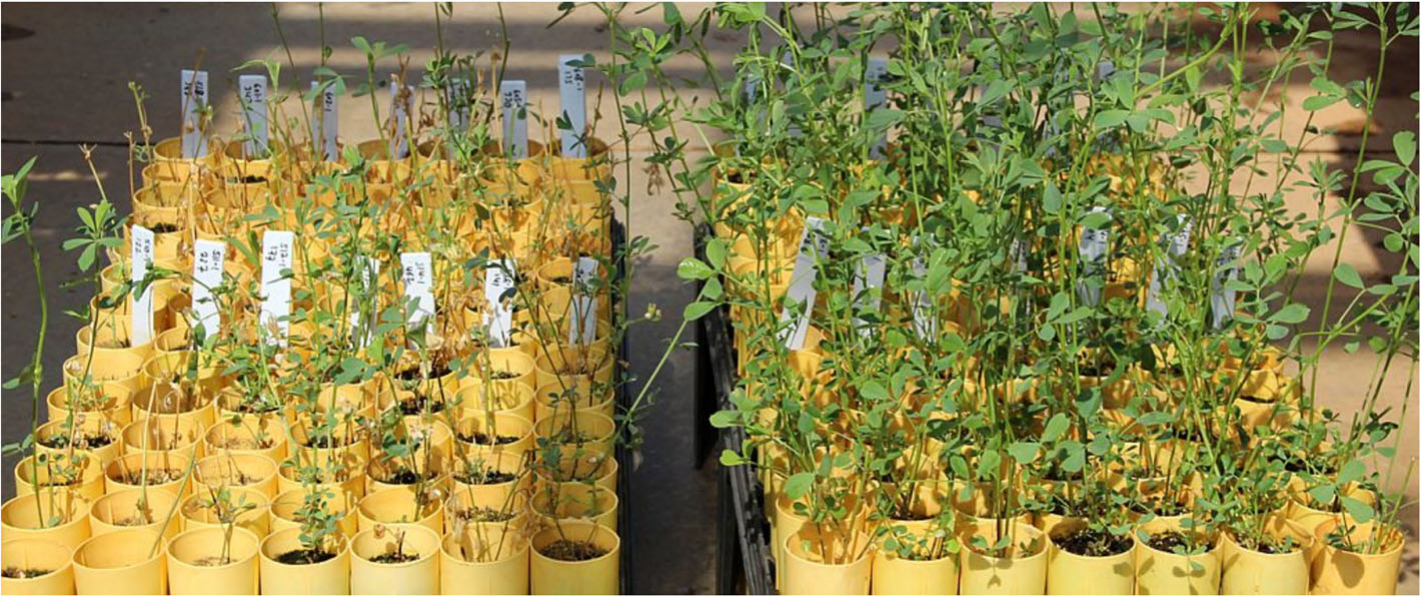
Regrowth pattern of indoor freeze-tested alfalfa plants from the treatment group (left) and the control group (right) after two weeks of freezing test. Some tested genotypes in the treatment group had good regrowth while some of them could not survive

The standard check seedlings in control and treatment sets of cultivars 5262 (WS = 2) and G-2852 (WS = 4) showed significant differences (*P* < 0.05) (Fig. 1) when the freezing environment was adjusted in such a way that the minimum testing temperature was -8 °C. The optimized protocol included a series of combinations of temperatures and durations of exposure. After acclimation, both treatment and control plant sets were maintained at 0 °C for 8 hr. Then, the control set was removed from the freezing chamber and transferred to normal growing conditions at 14 hr. of light (23 °C) and 10 hr. of dark (15 °C). Plants in the treatment sets were treated in such a way that the temperature was decreased by 2 °C/hr until it reached -8 °C. The plants were maintained for 90 min at -8 °C, then the temperature in the chamber was raised gradually by 2 °C/hr until it reached 2 °C. The treated plants were then transferred to the normal greenhouse condition for phenotyping.

### Phenotypic variation and distribution

**Fig. 2:**
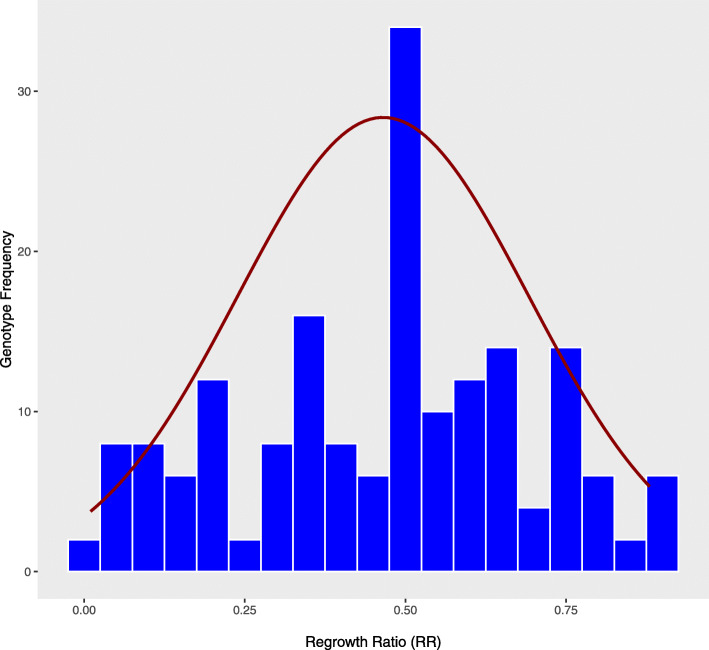
Distribution of mean of regrowth ratio (RR) computed for treated vs. a control group of plants among indoor tested alfalfa bi-parental (3010 x CW 1010) F_1_ progenies. The data is available for 179 progenies and the trait values exhibited near to normal distribution

Phenotypic variability was noticed among the F_1_ individuals for all four traits: freezing tolerance (FT), percentage survival (PS), regrowth ratio (RR), and biomass ratio (BR) (Figs. 1 and 2). All traits showed the nearly normal variability distribution within the population, where the highest number of samples had average trait values. Analysis of variance (ANOVA) for the traits like regrowth height and biomass assuming the traits values as a function of genotypes and replications showed that only genotypes have significant (*P* < 0.05) effects. We also noticed transgressive segregants for all four traits. The PS of cold-treated genotypes ranged from 7 to 100 %. The mean RR of surviving plants in treatment versus control ranged from completely sensitive (near 0) to almost completely tolerant (~ 1) genotypes (Fig. 2). Similarly, the mean BR of surviving plants in treatment and control sets ranged from 0.01 to 0.99. The average visual rating based FT of F_1_ plants varied from 1 to 4.9, indicating sufficient variation present in the F_1_ genotypes for freezing temperature tolerance. The genotypes with higher PS, RR, and BR, and lower FT were considered cold hardy genotypes. The PS showed a strong negative correlation (*r* = -0.91, *P* < 0.01) with mean FT, which means that the higher the % of the surviving clones, the better is the freezing tolerance of the genotypes (Table 1). The significant positive correlations (*r* = 0.55, *P* < 0.001) between PS and RR as well as PS and BR (*r* = 0.40, *P* < 0.01) indicated that the genotypes with higher % survival produced higher regrowth and subsequently higher biomass. Strong negative correlations were also observed between variables FT and RR, suggesting that the cold-sensitive genotypes had a low regrowth. Similarly, significant negative correlations (*r* = -0.46, *P* < 0.01) were obtained between BR and FT as with moderate correlation values (Table 1).

**Table. 1:** Correlations between different variables from indoor freezing tolerance test and winter hardiness data recorded under field conditions

	PS	RR	BR	FT	WH017JPC	WH-JPC	WH017BVL	WH-BVL
PS		0.55^***^	0.40^**^	-0.91^***^	-0.33^**^	-0.26^*^	-0.23^ns^	-0.19^ns^
RR			0.78^***^	-0.65^***^	0.19^ns^	-0.25^*^	-0.22^ns^	-0.25^ns^
BR				-0.46^***^	-0.25^*^	-0.28^*^	-0.20^ns^	-0.13^ns^
FT					0.36^**^	0.25^*^	0.23^*^	0.24^*^

WH017JPC = Correlation was computed using genotypes LS means for WH score recorded in 2017 at UGA JPC farm

WH-JPC = Correlation was computed using genotypes LS means for WH score recorded for overall (3 years) at UGA JPC farm

WH017BVL = Correlation was computed using genotypes LS means for WH score recorded in 2017 at UGA Blairsville farm

WH-BVL = Correlation was computed using genotypes LS means for WH score recorded for overall (3 years) at UGA Blairsville farm

*FT *Freezing tolerance, *PS *Percentage survival, *RR *Regrowth ratio, *WH *Winter hardiness, *BR *Biomass ratio

^*^P < 0.05, ^**^P < 0.01, ^***^P < 0.001, ^ns^ non-significant

We found a significant positive correlation (*r* = 0.36, *P* < 0.01) between mean FT and the least square (LS) mean of WH scores collected at the JPC field location in 2017 (WH017JPC). A significant positive correlation (*r* = 0.23, *P* < 0.01) was also observed between FT and WH data from Blairsville location (BVL) in 2017 (WH017BVL) (Table 1). Besides FT, other variables, such as PS, RR, and BR, from indoor testing also displayed significant correlations (*P* < 0.05) with WH data from the field (Table 1). However, we could not find significant correlations (*P* < 0.05) between field data for BVL and the variables PS, RR, and BR. However, the direction of the relationship between them was similar to that exhibited by the JPC field data. These relationships among the traits indicated the values of the phenotyping method used.

### QTL mapping

**Fig. 3:**
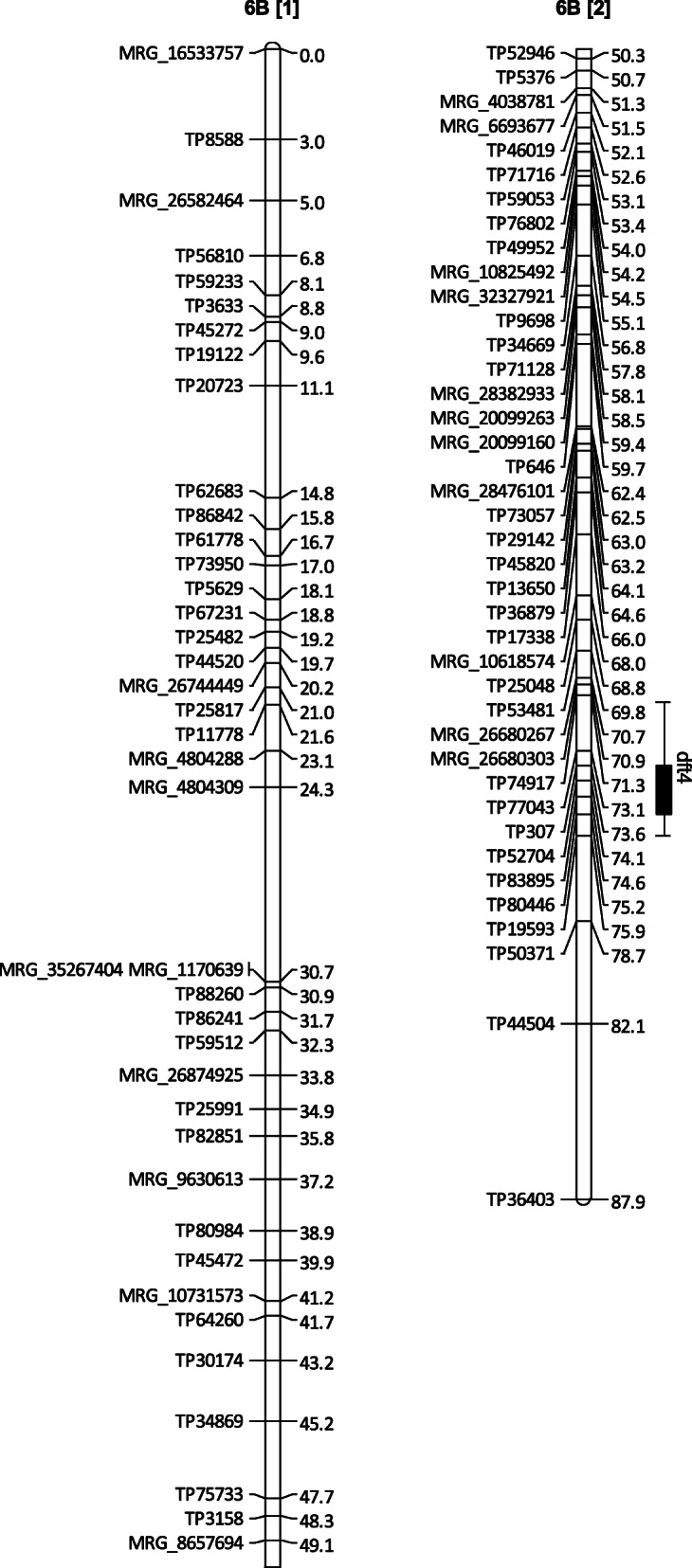
Linkage map of homolog 6B for the maternal parent (3010) where two maps 6B [1] and 6B [2] indicate two portions of a single linkage map 6B. The map shows a QTL (dft4) for freezing tolerance. The rectangle of the QTL bar represents an inner (1-LOD support) interval, and the line of the bar indicates an outer (2-LOD support) interval

As we generated two groups of relatively dense (1.5 cM/SNPs) linkage maps specific to each parent, we mapped QTL separately on them. In this study, we detected a total of 20 QTL for four traits (Table 2). The QTL that were detected on the linkage map of the dormant (d) parent (3010) were coded as ‘d’ followed by the trait name (in lower case abbreviation) and numbers. For example, dft1, dft2,…, dft5 represent different QTL detected on the dormant parent map for the freezing tolerance (Fig. 3). The QTL mapped on the non-dormant (n) parent linkage map were coded using the same approach just by replacing ‘d’ by ‘n’, e.g., freezing tolerance QTL of the non-dormant parent were named as nft1, nft2, and so on (Table 2).

**Table 2:** QTL for the traits related to cold temperature tolerance in an F_1_ pseudo-testcross (3010 × CW 1010) population mapped on the parent-specific linkage maps

Trait	Parent	QTL code	Chr	Peak Marker	Peak LOD	R^2^	Allele dir.	LSI (cM)	Flanking Markers
Freezing tolerance (FT)	3010	dft1	3C	TP49015	6.2	0.19	-	3.3 - 6.2	TP45927 - TP70292
		dft2	3C	TP49370	5.8	0.18	-	9.0 - 10.9	TP78263 - TP75378
		dft3	4B	TP65152	5.1	0.17	-	85.3 - 90.2	TP65152 - TP15305
		dft4	6B	TP52704	4.0	0.12	+	73.6 - 75.2	TP77043 - TP80446
		dft5	6D	TP4357	3.2	0.10	+	0 - 3	TP4357 - TP36795
	CW 1010	nft1	2A	TP48213	3.3	0.11	+	49.9 - 52.9	TP48213 - TP87635
		nft2	5B	TP50569	5.4	0.24	-	69.6 - 70.9	TP61799 - TP52871
		nft3^a^	5B	TP8562	6.7	0.29	-	72.6 - 74.5	TP47547 - TP81049
		nft4^b^	5B	TP80460	6.4	0.28	-	78.1 - 81.5	TP47971 - TP56384
% survival (PS)	3010	dps1	8B	TP55382	3.4	0.10	+	84.2 - 87.1	TP3723 - TP54987
	CW 1010	nps1^a^	5B	TP8562	4.5	0.15	-	73.1 - 73.8	TP8562 - TP81049
		nps2^b^	5B	TP80460	4.3	0.14	-	78.1 - 79.4	TP47971 - TP56384
		nps3	7A	TP13294	4.3	0.13	-	76.6 - 81	TP32322 - TP13294
		nps4	8A	TP8423	3.0	0.07	-	11.5 - 12	TP34845 - TP9964
regrowth ratio (RR)	3010	drr1	3C	TP49015	3.4	0.12	-	4.1 - 6.2	TP45927 - TP70292
		drr2	3C	TP18855	3.09	0.11	-	10.9 - 14.8	TP75378 - TP55943
	CW 1010	nrr1	4D	TP66640	3.5	0.13	-	37.8 - 41.9	TP36603 - TP65276
		nrr2	8D	TP2543	3.1	0.11	-	44.2 - 46.0	TP2543 - TP88682
biomass ratio (BR)	3010	dbr1	2B	TP49524	5.1	0.18	+	20.6 - 22.5	TP78533 - TP43844
		dbr2	5D	TP61232	3.4	0.12	+	23.6 - 28.3	TP66670 - TP70116

*Chr* Chromosome, *R*^2^ % of phenotypic variation explained by QTL, + The loci have positive effect, - The loci have negative effect, *LSI* 1- LOD support interval, *dir.* Direction, *cM* Centimorgan

^a^Overlapping QTL pair 1

^b^Overlapping QTL pair 2

Of five QTL detected for FT on 3010 linkage maps, two were on homolog 3 C, one on 4B, one each on 6B, and 6D (Table 2). Among the five FT QTL from the maternal parent, the QTL dft1 (R^2^ = 0.19) explained the highest phenotypic variation. Since we used only a single dose allele locus for linkage grouping that represents only a portion of the loci responsible for the trait in autotetraploid species, here we reported only the direction of allelic effects instead of actual additive effects. Of the five FT related QTL from the 3010 parent, only two QTL (dft4 and dft5) on chromosome 6 homologs (Table 2; Fig. 3) had positive effects for freezing tolerance while the other three were enhancing freezing sensitivity. We detected two QTL for RR (drr1 and drr2), two QTL for BR (dbr1 and dbr2), and only one QTL for PS (dps1) on the 3010 linkage maps (Table 2). Of the 10 QTL reported for the 3010 (winter-hardy) parent, five QTL exhibited favorable loci with positive impacts on the freezing tolerance-related traits (Table 2).

We identified four QTL for FT, four for PS, and two for RR on the linkage groups of CW 1010 (Table 2). For FT, we identified three of four QTL on 5B of CW 1010, where a QTL (nft3) explained the phenotypic variation up to 29 %. Also, two QTL for PS (nps1 and nps2) were reported in the same region on 5B for CW 1010. These five QTL: nft3, nps1 (overlapping), nft4, nps2 (overlapping), and nft2 on 5B of CW 1010 with negative effect suggested that the chromosomal segment is crucial for freezing sensitivity in alfalfa. We also observed two QTL for regrowth ratio (nrr1 and nrr2) for this paternal parent on chromosomes 4D and 8D (Table 2). Of the total 10 QTL identified for CW 1010 (cold-sensitive parent), only one QTL (nft1) had a favorable (+) effect for alfalfa freezing tolerance that explained only 11 % (R^2^ = 0.11) of the phenotypic variation. This output affirms the reliability of the trait value we used.

Some QTL identified in this experiment overlapped with genomic regions of WH-related QTL reported previously. The QTL nrr1 on chromosome 4D was detected in the same chromosomal region where the QTL ws10 was detected [17]. The QTL nrr2 reported here also overlapped with ws5 on chromosome 8D of CW 1010 parent [17]. Another QTL dbr1 of 3010 on chromosome 2B was identified in the proximal region where winter hardiness QTL wh15 and dormancy-related QTL dorm16 were detected [17]. The direction of the allelic effect of these QTL overlapped and matched in both phenotyping conditions. Also, in this study, we detected major QTL on various homologs of chromosomes 2, 3, 4, 5, 6, and 7. Another experiment also reported major winter injury-related QTL on linkage groups 2, 3, 4, 5, 6, and 7 [23]. These pieces of evidence support the genetic relationship between freezing tolerance and field cold survival. The tag sequences of flanking and peak markers of the QTL were provided (Supplementary File S1) can be used as potential markers for marker-assisted selection (MAS).

### *CBF* ortholog and relevant freezing tolerance QTL

Sequence alignment of a *CBF* gene (Supplementary File S2) of *M*. *truncatula* on alfalfa reference using the BLAST algorithm showed the alfalfa *CBF* orthologs exist around 75 Mb on chromosome 6 (Table 3). Since the alfalfa available reference has only eight chromosomes assigned [24], and it lacks sequence information for all four homologs of chromosome 6, we were not able to know if all homologs of chromosome 6 possess *CBF* genes. Also, we observed 91 % maximum identities when *M*. *truncatula CBF* orthologs aligned on alfalfa reference, suggesting that changes have occurred in the gene while evolving alfalfa from *M*. *truncatula*. A most interesting observation is that the *CBF* best BLAST hits occurred in the vicinity of the genomic region 75.72 to 75.77 Mb on chromosome 6, which suggests multiple genes of *CBF* families in the region.

**Table 3:** *M*. *truncatula CBF* sequence BLAST on *M*. *sativa* reference genome showed the following alignment status. The information was provided for BLAST alignment rate > 85 %

Subject Chromosome	Identities (%)	Alignment length (bp)	Total gaps	Query start	Query end	Subject start	Subject end	Score	E-value
6	91	1394	21 (2 %)	243	1626	75,778,859	75,780,241	1823	0
6	91	1142	19 (2 %)	78	1212	75,731,872	75,733,001	1504	0
6	89	1157	14(1 %)	78	1229	75,770,149	75,769,002	1437	0
6	88	1142	16(1 %)	78	1212	75,728,433	75,729,565	1358	0
6	86	1219	23(2 %)	3	1212	75,766,951	75,768,155	1315	0

When we aligned the tag sequences (~ 60 bp) of markers related to the cold tolerance QTL: dft4 (chromosome 6B) and dft5 (chromosome 6D), we observed the dft4 related markers tag sequences best BLAST hit occurred around 120 Mb and dft5 related markers tag sequences best aligned around 10 Mb on chromosome 6. There could be multiple reasons behind the non-overlapping of the positions of *CBF* ortholog and dft4 markers, such as we might have missed detecting *CBF* loci since we only used SDA SNPs. Because of the lack of sequences of all four homologs of alfalfa in the reference genome, we have currently no way to know exactly where the *CBF* resides on the homologs and how many different copies of the gene are there. Also, a very short tag sequence (~ 60 bp) alignment could be questioned. Nevertheless, the recently published reference genome of alfalfa and the available cloned gene of *M*. *truncatula* provided us an opportunity to determine the potential location of alfalfa orthologs of a cold tolerance gene.

### Indoor screened breeding material

After consecutive cycles of freezing tolerance testing, selection, and polycrossing, we developed 177 half-sib families. At the University of Georgia (UGA) research farm at Blairsville, GA, these lines were screened in the field for cold hardiness together with winter survival and fall dormancy standard checks. The fully established plants showed better winter survival (40–70 % stand count) after the 2019–2020 winter. Nonetheless, the winter survival checks exhibited 16–80 % survival. The winter survival checks: ZG9830 and WL325HQ showed up to 80 % of survival whereas CUF 101 showed only 16 % survival. Further, the fall dormancy (FD) check UC-1465 died completely after the winter frost, whereas another FD check ABI 700 (semi-dormant) had only a 10 % survival. These observations indicated that better survival of some winter survival checks is probably due to their dormant nature. Since the winters in Blairsville, GA are mostly severe with 29.3 °F (-1.5 °C) average low temperature in January (https://www.weather-us.com/en/georgia-usa/blairsville-climate), the non-dormant alfalfa germplasm that tolerates the harsh winter with better cold tolerance ability constitutes an improved genetic resource for cold-hardy alfalfa cultivar development.

## Discussion

### Alfalfa indoor screening for freeze tolerance

The indoor freezing tolerance selection method we reported here is relatively faster and cheaper than field selection as it takes only a few hours in a freezer after acclimation. All the steps are conducted in a controlled environment and hence the freezing test can overcome the difficulties associated with the field phenotyping mainly environmental variability and the unpredictable winters. Past studies have established that alfalfa winter hardiness in the field is influenced by the ability of genotypes to tolerate and survive freezing [7], suggesting an optimized freezing test is valuable. This indoor FT selection also enables the screening of a large number of accessions at the same time; normally, we tested a set of 196 (98 × 2) clones grown in cones that included both control and treatment groups. Nonetheless, the indoor-selected alfalfa may also require few cycles of field selection before the release as a cold-tolerant cultivar. A previous study indicated that indoor selected alfalfa plant progeny showed an increase in freezing tolerance up to 5˚C and superior winter survival [3].

**Fig. 4:**
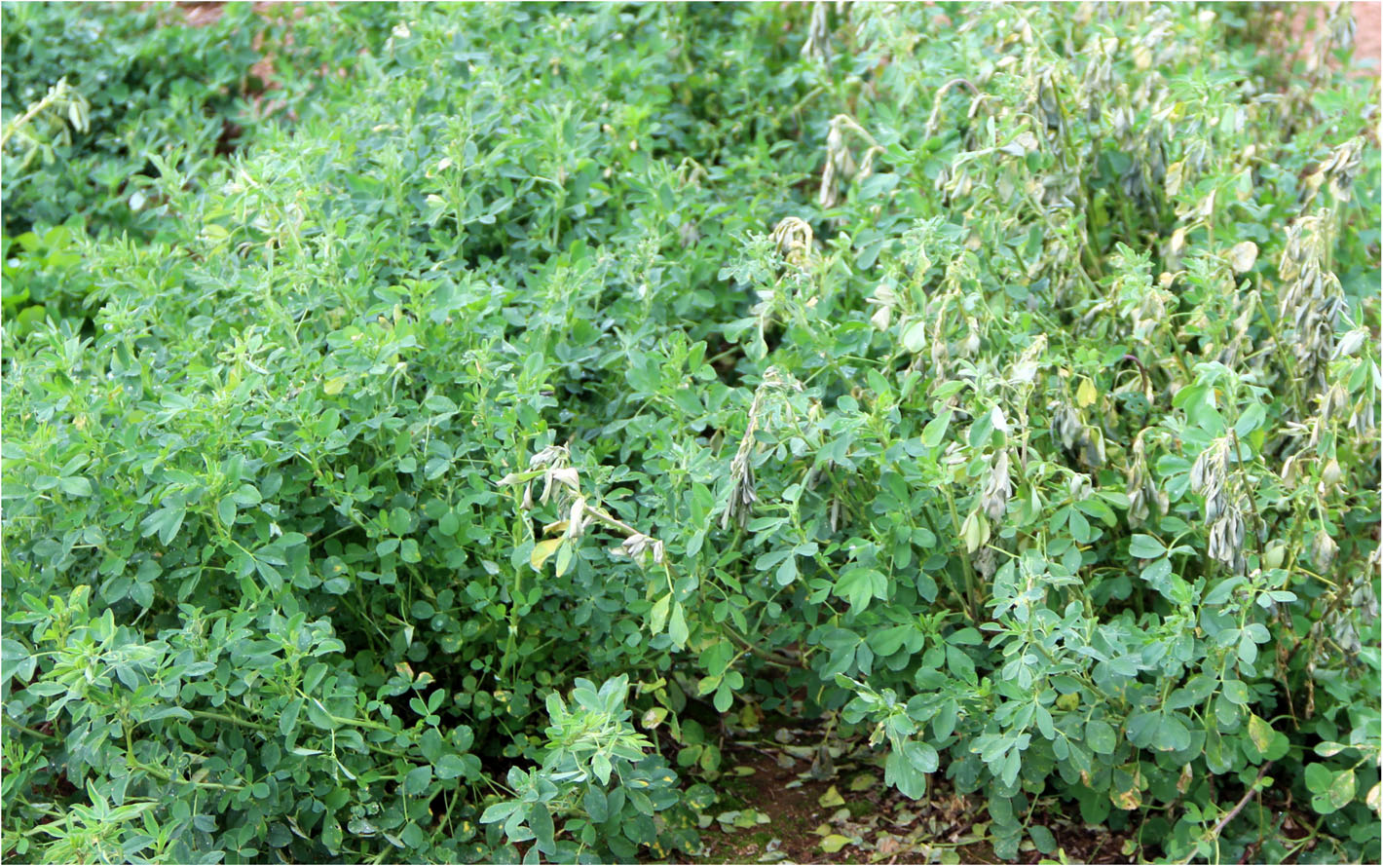
Winter frost tolerant (left) and sensitive (right) alfalfa F_1_ progenies. The sensitive alfalfa has very distinct frost damage symptoms. The image was taken after frost occurrence in the first week of March 2017 at Watkinsville, GA. Before the frost, the winter was mild, and the alfalfa had gained sufficient regrowth

In mild winter areas where the winters have occasional frost and fluctuating temperatures, the condition can be damaging to perennial forages such as alfalfa with the non-dormant types being especially more vulnerable. When winters are warm with a temperature near 13 °C, over-wintering alfalfa breaks dormancy and starts new growth with elongated crown buds  (http://www.canr.msu.edu/uploads/resources/pdfs/e2310.pdf), the phenomenon is known as deacclimation [25]. The process depletes alfalfa root reserves, which eventually makes the plant susceptible to low temperatures (http://www.canr.msu.edu/uploads/resources/pdfs/e2310.pdf). Thus, the sensitive genotypes fail to reacclimatize when frost returns in late winter and early spring (Fig. 4). Therefore, it seems that the efficiency of the freezing test would be enhanced by the freezing of the already tested (deacclimated) clones one more time. In other words, a simulated environment that involves a series of acclimation-deacclimation-reacclimation processes could be ideal for future freezing tests in alfalfa.

### Clonal variations

One of the challenges encountered in the alfalfa indoor cold test was to generate identical clones by stem cuttings. The clonal variation is not unusual and such variation among clones of stem cuttings of the same genotype is commonly experienced in alfalfa. Perhaps the uncontrolled source of variability is attributable to the vigor of establishment and the interaction with the environment (watering, location in the growth chamber, etc.) (Yves Castonguay, personnel communication, 2016). This effect can be minimized by making multiple clonal propagules for each genotype and using vigorous clones of uniform size for freezing treatment. When resources are not limited, producing a large number of cuttings and testing more uniform clones could be effective to avoid the uncontrolled source of variability among clones. In this study, since the tested clones were selected carefully for their uniformity, there were no significant variations (*P* < 0.05) among the surviving replicated clones of a genotype for the traits like regrowth and biomass. However, we found different levels of survival (PS) within the clones of a single genotype after exposure to freezing, suggesting that PS is a quantitative trait and varies from genotype to genotype. The phenotypic variation present in clones, which is also known as somaclonal variation, could be the result of other factors such as epigenetic changes [26].

### FT and WH relationship

The moderate positive correlation (*r* = 0.23 to 0.36) observed between indoor cold screening and field WH rating (Table 1) indicated that WH selection in the field can be accelerated by screening alfalfa in the freezing chambers. Some past studies also reported similar information. Brouwer et al. [7] found a positive correlation (*r* = 0.34 to 0.58) between freezing injury and winter injury in field conditions and suggested that freezing tolerance and WH are potentially controlled by some common genetic mechanism. A breeding population developed using indoor selected parents accelerated the breeding process [3]. For instance, superior freeze-tolerant cultivars like Apica (ATF0) and (ATF5) were developed using recurrent selection for up to five cycles [27]. The results from this study and past reports indicated that an indoor freezing test can be useful in indirectly selecting alfalfa winter hardiness.

Winter hardiness is a broad term that refers to the plant’s ability to withstand harsh winters, which encompasses freezing temperature, diseases, high moisture level, ice formation, and frost-heaving (https://extension.umaine.edu/publications/2272e/) [28]. Therefore, selecting only based on FT represents only a part of the spectrum of variables and breeders also need to test the selected freezing tolerant plants in the field. For instance, different levels of carbohydrate accumulation in the alfalfa crown after artificial freezing and natural hardening were reported [29]. Artificial freezing tolerance was related to the accumulation of sucrose, stachyose, and raffinose and decreased levels of glucose, fructose, and starch. In contrast, alfalfa natural hardening triggered the accumulation of raffinose and stachyose and was less relevant to sucrose accumulation [29]. Perhaps, these physiological and molecular factors are behind the moderate relationship between indoor FT and field WH we have seen in the present study.

### FT QTL, candidate gene and future direction

In this experiment, we found six favorable QTL (Table 2) that may have the potential to be used for MAS for alfalfa freezing tolerance as they individually explained more than 10 % of the phenotypic variance (R^2^ ≥ 10). Several other QTL with negative effects on freezing tolerance could be an interesting region to investigate the molecular basis of FT in alfalfa. Freezing tolerance in plants is regulated by a complex network of genes [30]. As an autotetraploid that still lacks complete homologs level reference genome, genomic studies of alfalfa are relatively complicated. Also, the species exhibits tetrasomic inheritance with a complicated segregating pattern so that including genome-wide marker information is difficult. Finding certain QTL using pseudo-testcross markers does not account for all loci governing the traits of interest. Because the crop is allogamous, developing other efficient QTL mapping populations such as recombinant inbred line (RIL) is impossible in alfalfa. Also, there is no established method to produce doubled haploid (DH) population in this autopolyploid species as in other species [31] which could be used to map the stable QTL. Therefore, any small information corresponding to the QTL and candidate gene for traits of interest in alfalfa is valuable in this genomic era.

Aligning *M*. *truncatula CBF* sequence and the presence of a freezing tolerance QTL (dft4) nearby on chromosome 6 indicated that more investigations are required in this candidate region. Past reports indicated that *M. truncatula* has a set of *CBF* genes on the long arm of chromosome six [32]. Expression analysis of *CBF*-related genes from *M*. *trunctatula* in alfalfa indicated that the genes (*MsCBFl-17* and *MsCBFl‐18*) are potential functional homologs of *CBF3* [33]. The *CBF* homologs and gene group were located not only on *M*. *trucatula* chromosome 6, but also on chromosome 5 [33, 34] on which we also detected several QTL associated with freezing sensitivity and a favorable QTL (dbr2) (Table 2). As we observed several freezing sensitivity-related QTL, the genomic regions may also harbor cold regulated (*COR*) genes that influence the expression of *CBF* in cold susceptible alfalfa.

Furthermore, the multiple QTL for FT and WH [17] with low to moderate R^2^ indicated that genomic selection (GS) could be a viable option to improve these traits. GS is becoming an effective method for the rapid selection of superior genotypes for cold hardiness improvement in different crops [35, 36]. Implementation of GS in forages and legumes for enhancing genetic gain through reduction of average cycle time and increase of selection accuracy has been perceived as a beneficial selection approach at present and for the future [37]. For instance, Poudel et al. [38] described GS as an effective approach to developing cold-tolerant cultivars of bioenergy crop switchgrass for northern latitudes. The potential application of GS in alfalfa has been described [39, 40].

Since the QTL identified in this study explained at least 10 % variance, integrating these QTL as fixed effect covariates in a GS model could be an effective approach to enhance alfalfa cold tolerance. However, the validation of QTL to multiple environments and diverse germplasms must be warranted before using them as covariates. Several past reports recommended that the inclusion of major QTL or genes in the GS models augmented the prediction accuracy of the model in different crop species [41–44]. Bernardo, R.(2013) reported if the percentage variance explained by the major gene is > 10 %, then using it as a fixed effect covariate improves prediction accuracy in genomewide selection. We also believe that using major cold tolerance QTL of alfalfa as a fixed effect in future GS studies would be effective for the better predictive ability of the model. As GS is based on whole-genome markers, utilizing QTL in the model most likely enables the reduction of the number of the required markers from the whole set. Further, the indoor phenotyping approach described here can be useful in phenotyping the training population designed for GS for alfalfa winter hardiness selection.

## Conclusions

In this study, we reported an indoor approach for rapid screening of alfalfa freezing tolerance (FT) which is useful for accelerating the breeding for winter hardiness (WH) in the field. Since FT and WH are moderately correlated, we suggest the indoor selection of alfalfa germplasms before taking them to the field for better winter survival selection. We identified 20 QTL for FT-related traits in the population tested. The most favorable QTL were located in the proximity of chromosome six, where the crucial cold tolerance gene family (*CBF*) has been reported for the alfalfa closest relative *M*. *truncatula*. The validation of these QTL in various genetic backgrounds will be warranted before adopting them in MAS to ensure the loci are stable and repeatable across populations and environments. However, some of the favorable QTL that explained 10 % or more of the phenotypic variance could be tested directly in MAS. We suggest that genomic selection of alfalfa with the incorporation of validated QTL with major effects as covariates would be ideal to enhance a quantitative trait like freezing tolerance.

## Methods

### Plant materials

The plant materials used in this experiment include two alfalfa parent cultivars, their F_1_ progenies, and winter survival checks. The commercial certified seeds of alfalfa cultivar CW 1010 and 3010 were obtained from Alforex Seeds (Woodland, CA, USA) and BrettYoung (Winnipeg, Manitoba, Canada), respectively. The CW 1010 has the fall dormancy level ten (FD = 10) and the cultivar 3010 has FD level two (FD = 2). The seeds were collected in accordance with the national and internal guidelines. The winter survival of the cold-tolerant parent 3010 is two (WS = 2), whereas the WS rating of CW 1010 has not been defined (https://www.alfalfa.org/pdf/2021_Alfalfa_Variety_Leaflet.pdf). From field observations, CW 1010 has shown extensive damage in winter and has been considered as one of the winter susceptible alfalfa cultivars. More information about these varieties is found in NAFA variety ratings (https://www.alfalfa.org/pdf/2021_Alfalfa_Variety_Leaflet.pdf) and the details about the varieties can also be searched in the database (https://www.alfalfa.org/varietyratings.php). The check varieties were obtained from the National Plant Germplasm System (NPGS). We screened the alfalfa pseudo-testcross F_1_ [3010 (♀) x CW 1010 (♂)] population for freezing temperature response. The maternal parent ‘3010’ is a dormant type alfalfa cultivar with relatively higher winter survival whereas CW 1010 is non-dormant and cold-sensitive. The mapping population development and field experiment were described previously [17, 45, 46]. Briefly, hand pollination was performed to develop F_1_ progenies and the genotyping of F_1_ was performed with 3 SSR markers to confirm they were hybrid. The population was then grown in two locations, Blairsville and Watkinsville research farms of the UGA using three clonal replications in a randomized complete block design (RCBD). This study focused on freeze testing of alfalfa clones of the mapping population using a walk-in freezer (ESPEC North America, Inc.). Twenty clones per genotype were generated by stem-cuttings of 184 F_1_ progenies and parents. The clones were grown under normal growing conditions and management in the greenhouse. Prior to cold acclimation, twenty clones were divided into two sets, one for control and the other is the treatment group. Together with the experimental mapping population, we also grew the alfalfa winter survival (WS) check cultivars recommended by NAAIC to optimize the freezing test. The checks included the cultivars ZG 9830 (WS = 1), 5262 (WS = 2), WL325HQ (WS = 3), G-2852 (WS = 4), Archer (WS = 5), and Cuf 101 (WS = 6), where WS ‘1’ and ‘6’ indicated extremely winter-hardy and non-winter-hardy, respectively (https://www.naaic.org/stdtests/wintersurvivalnew.pdf).

### Indoor phenotyping

For the check cultivars, we tested seeded plants while for the population, we tested vegetative clones. The check seeds were sown in 14 cm tall (Ray Leach, SC10) cone-tainers filled with farm soil and 5 cm depth of the Fafard germ mix (Fafard, MA) at the top of the cone. The Cecil sandy loam type farm soil falls under soil class fine, kaolinitic, thermic Typic Kanhapludults as described previously [47]. The seeded cones were placed in RL98 trays and the seedlings were grown in the greenhouse for 6–8 weeks. Then, the plants were transferred to an acclimation chamber at a temperature of 4 °C for 3 weeks. The chamber was adjusted to 8/16 (light/dark) hours period, and 70 % relative humidity (RH). The plants were watered weekly and fertilized with Hoagland’s nutrient solution once during the acclimation period. The plants in treatment and control sets were randomized so that both sets have uniform phenotypic distributions. We removed any cryptic clones that look phenotypically different than their sister clones. Various combinations of cold temperatures and times of exposure were tested to optimize the freezing trial until significant differences (chi-square test with *P* = 0.95) were observed between samples of the checks 5262 (WS = 2) and G-2852 (WS = 4), as described previously (https://www.naaic.org/stdtests/wintersurvivalnew.pdf). After the freezing test, the plants were moved to normal greenhouse conditions. The top portion of the plants in both the control and the treatment groups were then clipped leaving two nodes above the crown to allow regrowth. After two weeks from treatment, the data were recorded as survival percentage, regrowth height, and visual rating based on freezing tolerance on a scale of 1–5, with 1 being the most freezing tolerant and 5 being the most freezing sensitive. The criterion used for freezing tolerance rating had a slight modification in the bases used for winter survival rating (https://www.naaic.org/stdtests/wintersurvivalnew.pdf). We scored 1 for genotypes with no injury and uniform regrowth whereas 5 was given to heavily injured and barely survived genotypes. We also recorded the biomass of tested samples after three weeks of the freezing test. Four traits, visual rating based freezing tolerance (FT), percentage survival (PS), treatment vs. control regrowth ratio (RR), and biomass ratio (BR) were recorded. 

$$mean \; regrowth\; ratio \;\left(RR\right)= \frac{mean \;regrowth\;of\;cold\;treated\;clones\;of\;a\;genotype}{mean\;regrowth\;of\;acclimated\;control\;set\;clones\;of\;the\;genotype}$$

### Relationship between FT and WH

Phenotypic correlation between FT traits from indoor screening and WH scores from the field was computed using the PROC CORR procedure in SAS 9.4 (SAS Institute Inc.). In the 2016/2017 winter, Georgia experienced mild weather but the early spring frost that occurred in the first week of March caused severe damage in the new regrowth of the winter. Therefore, we compared the FT with WH field data obtained for the season 2016/2017 from both locations and combined environments (Table 1).

### GBS and marker identification

GBS library preparation, Illumina sequencing, and data processing steps were clearly described previously [17]. In summary, alfalfa mapping population and parental samples DNA were extracted from the freeze-dried tissues with minor modification of the method described [48]. Then, two 96-plexed GBS libraries were constructed using ApeKI DNA digestion and following the method described in [49]. The libraries were sequenced at Georgia Genomics and Bioinformatics Core (GGBC) using Illumina NexSeq (150 Cycles) 75 PE High Output flow cell with four lanes. We processed the sequencing data using two different pipelines: Universal Network-Enabled Analysis Kit (UNEAK) [50] and GBS-SNP-CROP [51] for single nucleotide polymorphisms (SNP) identification. With the former pipeline, we only used the forward (R1) reads while with the latter we used both forward (R1) and reverse (R2) reads.

### Genetic linkage and QTL mapping

The single nucleotide polymorphism (SNP) markers identified using GBS were filtered for missing (removed < 30 % missing) data. Then, we filtered again for single-dose allele (SDA) loci polymorphic (Aaaa x aaaa) to a unique parent as described [49]. The SDA SNPs segregating ratio 1:1 (AB:AA) was tested using chi-square test (α = 0.05). Since, the species exhibits tetrasomic inheritance, utilizing all markers with the available computational resources was not possible. Therefore, we only used markers that were segregating as in pseudo-testcross progenies and the strategy is called pseudo-testcross strategy. Then, we constructed 32 linkage groups for 8 chromosomes and each of four homologs separately for the maternal and paternal parent using regression mapping on JoinMap 5.0, where we used Kosambi mapping function and minimum independence LOD value 10 for marker grouping [17, 52]. The 32 linkage maps specific to the maternal parent retained 1837 SDA SNPs, whereas paternal linkage maps retained a total of 1377 SNPs. The average marker densities for linkage maps of each parental type were 1.5 cM/SNP. The chromosome of corresponding linkage groups was assigned based on the alignment of marker tag sequences on *M*. *truncatula* reference V4.1 [17]. We mapped the freezing tolerance-related traits on the linkage maps using Windows QTL Cartographer version 2.5. The QTL mapping was conducted with the composite interval mapping (CIM) method [53]. The LOD score specific to the trait was computed using 1000 permutations at a genome-wide threshold of *P* ≤ 0.05 [17]. The genotypic means of all four traits were used as trait values for QTL mapping.

### Genomic analysis of candidate QTL

As an alfalfa genome has recently been published [24], we conducted sequence alignments of a cold-related gene C-repeat binding factor (*CBF*) of *M*. *truncatula* on alfalfa reference (Table 3), alfalfa sequence has not been cloned. The alignment allowed us to find *CBF* orthologs region on the alfalfa genome and relate it to the QTL we identified for cold tolerance. We also aligned tag sequences of markers under QTL regions, which most likely represent loci of *CBF* gene families, on alfalfa reference. However, the tag sequence was only around 64 bp in length (Supplementary File S1).

### Developing breeding materials

The freezing tolerant genotypes we selected in the study were deemed as source germplasms for winter-hardy alfalfa cultivar development. To begin the breeding and selection process, we transplanted the freeze-tolerant F_1_ plants in the greenhouse and performed polycrossing among the full sibs using bee-mediated pollination. The sib-mated F_2_ seeds were bulk harvested and germinated in the greenhouse. The seedlings were then screened for freezing tolerant genotypes using a constant freezer environment. The survival half-sibs were then transplanted in bigger containers and intermated. Then the F_2_ derived half-sib progeny seeds were harvested separately for each parent and germinated. Then the seedlings of these freeze-tested parents were grown in Blairsville GA in spring 2019 for the evaluation of their field performance. We evaluated the survival rate of the half-sib progenies after 2019/2020 winter to assess the winter hardiness ability of the plants.

## Supplementary Information

**Additional file 1: Supplementary File S1.** Tag sequences for flanking and peak markers identified within the QTL regions. Two variant alleles for each SNP were denoted as ‘query’ and ‘hit’.

**Additional file 2: Supplementary File S2.** Fasta sequence of *M*. *truncatula* cold tolerance related gene dehydration-responsive element-binding protein 1C (also known as *CBF5*) obtained from the National Center for Biotechnology Information (NCBI).

## Data Availability

The raw reads from genotyping-by-sequencing can be found at NCBI SRA under the accession number SRP150116 and with the link https://www.ncbi.nlm.nih.gov/sra/SRP150116. The GBS barcode key file required to demultiplex the SRA deposited fastq sequence files can be found at Dryad digital repository (10.5061/dryad.1g1jwstwv).

## Abbreviations

BR: Biomass ratio
*CBF*: C-repeat-binding factor
Chr: Chromosome
FT: Freezing tolerance
FD: Fall dormancy
GBS: Genotyping-by-sequencing
MAS: Marker-assisted selection
PS: Percentage survival
QTL: Quantitative trait loci
RR: Regrowth ratio
SNP: Single nucleotide polymorphisms
WH: Winter hardiness
WS: Winter survival
