# Supplementary material for: Mapping freezing tolerance QTL in alfalfa: based on indoor phenotyping
Source: BMC Plant Biol. 2021 Sep 6;21:403. doi: 10.1186/s12870-021-03182-4 (PMC8419964; doi:10.1186/s12870-021-03182-4)
Supplement: Supplementary file 1 — Additional file 1: Supplementary File S1. Tag sequences for flanking and peak markers identified within the QTL regions. Two variant alleles for each SNP were denoted as ‘query’ and ‘hit’. [file 12870_2021_3182_MOESM1_ESM.docx]

**Supplementary File S1.** Tag Sequences for flanking and peak markers reported within QTL that were mapped on maternal parent 3010. Two variant alleles for each SNP were denoted as ‘query’ and ‘hit’.

**Chromosome 2B**

>TP43844_query

CAGCTTGTTACATAGGATTAACTATTTCAACCAAATCTACAAACTTTCCTTCAAAATCTCTGTG

>TP43844_hit

CAGCTTGTTACATAGGATTAACTATTTCAACCAAATCTACAAACTTTCTTTCAAAATCTCTGTG

>TP49524_query

CTGCAAGACATGACAAATCGATTACAAGTATTTGTTTGTGGTTACATTACTCATAATTCTACTT

>TP49524_hit

CTGCAAGACATGACAAATCGATTACAAGTATTTGTTTGTGGTTACTTTACTCATAATTCTACTT

>TP78533_query

CTGCTATGGAAATCCCAAAAAGTATAGAAGAGAAGAAGCCGCCTTAAATGTTTCTCACATATTC

>TP78533_hit

CTGCTATGGAAATCCCAAAAAGTATAGAAGAGAATAAGCCGCCTTAAATGTTTCTCACATATTC

**Chromosome 3C**

>TP18855_query

CAGCATTGTCGTTGTTTTGATACGGTGCCACAGAGACTCTATTCCAAAATTGCATCCCATATTT

>TP18855_hit

CAGCATTGTCGTTGTTTTGATACGGTGCCACAGAGACTCTGTTCCAAAATTGCATCCCATATTT

>TP45927_query

CTGCAAAAGGTATCAACGTAGAAGCGAAGCAAGCCAATATGTTAATGAAGTTGTCAACCTCACA

>TP45927_hit

CTGCAAAAGGTATCAATGTAGAAGCGAAGCAAGCCAATATGTTAATGAAGTTGTCAACCTCACA

>TP49015_query

CTGCAACTCATTAACCATCTACTGAACGTAATTAACCGCGATTTTGAAAGTCATTCACCAAGAT

>TP49015_hit

CTGCAACTCATTAACCATCTACTGAACGTAATTAACCGCGATTTTGAAAGTCATTCACCATGAT

>TP49370_query

CTGCAAGAAACATCAACATAAACTCAACAAAGTCACTAAACCAAACAACGACTCTTCGTTTGAA

>TP49370_hit

CTGCAAGAAACATCAACATAAACTCAACAAAGTTACTAAACCAAACAACGACTCTTCGTTTGAA

>TP55943_query

CTGCAGAACAAAAATCCAATTACAAGCCAAAAACAGAACACAACTAATTTACGATTTACTGATA

>TP55943_hit

CTGCAGAACAAAAATCCAATTACAAGCCAAAAACAGAACACAACTAATTTATGATTTACTGATA

>TP70292_query

CTGCCTATGAAACACACAGACACTGACACGACACATTGACACTCATAATAATTTCTGAAAATGA

>TP70292_hit

CTGCCTATGAAAGACACAGACACTGACACGACACATTGACACTCATAATAATTTCTGAAAATGA

>TP75378_query

CTGCTAAAGCAAAAACAAGAGGAGGATCCCCGTCTTTGTCCAAGACATCAACATTTGCCTCTGG

>TP75378_hit

CTGCTAAAGCAAAAACAAGAGGAGGATCCCCGTCTTTGTCCAATACATCAACATTTGCCTCTGG

>TP78263_query

CTGCTATCATTACTCAGTTCGGGCTGTTAATGTGGATCTGCGTTTCTATTTCTCGTTTGCATCC

>TP78263_hit

CTGCTATCATTACTCAGTTCGGGCTGTTAATGTGGATCTGCGTTTCTTTTTCTCGTTTGCATCC

**Chromosome 4B**

>TP15305_query

CAGCATATCCGGCAACGTTGAATCCATTCACAGCGGCACCGTTTGTGGGCGTTGATGGTGCTTT

>TP15305_hit

CAGCATATCCGGCAACTTTGAATCCATTCACAGCGGCACCGTTTGTGGGCGTTGATGGTGCTTT

>TP65152_query

CTGCCACATTTTGAACTCCAGAGGTTGAGTGTTTGCGCTCATCAACCAATGGGAAATCATGTTG

>TP65152_hit

CTGCCACATTTTGAACTCCTGAGGTTGAGTGTTTGCGCTCATCAACCAATGGGAAATCATGTTG

**Chromosome 5D**

>TP61232_query

CTGCATGACGACGGTAAACTGCCAACATGCAATCCAATTTTCTATTGAAAGAGGTTTTCCAAGA

>TP61232_hit

CTGCATGACGACGGTAAATTGCCAACATGCAATCCAATTTTCTATTGAAAGAGGTTTTCCAAGA

>TP66670_query

CTGCCATCTCTGAGATTATTGGAAGACTTAAAAAGGGTTGCCTTCTCTTCCTCTTTTTCTTCAG

>TP66670_hit

CTGCCATCTCTGAGATTATTGGAAGACTTATAAAGGGTTGCCTTCTCTTCCTCTTTTTCTTCAG

>TP70116_query

CTGCCTACACCGAATTCTCAAACTCCTCAAGAGGTTTGCATCTATACAATATAATGTTGTTTCA

>TP70116_hit

CTGCCTACACCGAATTCTCAAACTCCTCAAGAGGTTTGCATCTATACAATTTAATGTTGTTTCA

**Chromosome 6B**

>TP52704_query

CTGCACAACAGTTGTAATATTCACTACGTACATACACGAATATAACATTGAAAAAACCAGCTTC

>TP52704_hit

CTGCACAACAGTTGTAATATTCACTACGTACAAACACGAATATAACATTGAAAAAACCAGCTTC

>TP77043_query

CTGCTACTGGTGGTTTTCTTGCTATGCGTCAGGGTTTAGGTGCTTCGGCGCGATCGGCGGCGTT

>TP77043_hit

CTGCTACTGGTGGTTTTCTTGCTATGCGTCAGGGTTTAGGTGCTTCGGCGCGGTCGGCGGCGTT

>TP80446_query

CTGCTCCGAATCTTTGCACCTTTACTTTTAATGGTACTCTTGATAAGAAAATATATGAGAGTGG

>TP80446_hit

CTGCTCCGAATCTTTGCACCTTTACTTTTAATGGTACTCTTGATCAGAAAATATATGAGAGTGG

**Chromosome 6D**

>TP4357_query

CAGCAACTTTTATCATATCATCCCAAACCATGCTTCCTTCTTCAAGTGCATCTGAAACCTTTGG

>TP4357_hit

CAGCAACTTTTATCATATCATCCCAAACCATGCTTCCTTCTTCAAGTGCTTCTGAAACCTTTGG

>TP36795_query

CAGCTCCGGTAATTCCTCTCCAAAGGCTCTCCACCAAACTGATGGAATCTCATTACGAGCTGTC

>TP36795_hit

CAGCTCCGGTAGTTCCTCTCCAAAGGCTCTCCACCAAACTGATGGAATCTCATTACGAGCTGTC

**Chromosome 8B**

>TP3723_query

CAGCAACGACAACAACAACAAACATCCAACTTACCACGGAGTAAGAAAACGAAACTGGGGAAAA

>TP3723_hit

CAGCAACGACGACAACAACAAACATCCAACTTACCACGGAGTAAGAAAACGAAACTGGGGAAAA

>TP54987_query

CTGCACTAGATGAACTGCTACTCGGTGCTCGCAAAACCCACAAGAATTTTTTATCAGTCAATTC

>TP54987_hit

CTGCACTAGATGAACTGCTACTCGGTGCTCGCAAAACCCACAATAATTTTTTATCAGTCAATTC

>TP55382_query

CTGCACTGTATTAAACTTAGTTATCTCTATCATTTCTAATGTATCTTGTTGAAATTTGGGGCCT

>TP55382_hit

CTGCATTGTATTAAACTTAGTTATCTCTATCATTTCTAATGTATCTTGTTGAAATTTGGGGCCT

**Tag Sequences** for flanking and peak markers reported within QTL that were mapped on paternal parent CW 1010. Two variant alleles for each SNP were denoted as ‘query’ and ‘hit’.

**Chromosome 2A**

>TP48213_query

CTGCAACAGATTCGTGGTGATGTTAAGGAGCTTAGTGCTGTTAGACAGGATTTGTCTGGTCAAG

>TP48213_hit

CTGCAACAGATTCGTGGTGATGTTAAGGAGCTTAGTGCTGTTAGGCAGGATTTGTCTGGTCAAG

>TP87635_query

CTGCTTGATCAACGTATTCTGATAATCTGCTCCAAAGGCCTGCCTCGAGCCAGTCCTCACGTGC

>TP87635_hit

CTGCTTGATCAGCGTATTCTGATAATCTGCTCCAAAGGCCTGCCTCGAGCCAGTCCTCACGTGC

**Chromosome 4D**

>TP36603_query

CAGCTCCATTCTAAGAATCGTCCTCGGTTGAAATATGTATTTCGCACCTTCAACCTACAGTTTA

>TP36603_hit

CAGCTCCATTCTAAGAATCGTCCTCGGTTGAAATATGTATTTGGCACCTTCAACCTACAGTTTA

>TP65276_query

CTGCCACCCCGGTTTTTGCCAAAAATACACTTTGGAACTTCAACAAAACCCTGGTGGTAACCAT

>TP65276_hit

CTGCCACCCGGGTTTTTGCCAAAAATACACTTTGGAACTTCAACAAAACCCTGGTGGTAACCAT

>TP66640_query

CTGCCATCGTTGAAACAATTGTTGGTGGTTTTTTGTGCTTCACGTTTAGGGGTTAAGACTTGTA

>TP66640_hit

CTGCCATCTTTGAAACAATTGTTGGTGGTTTTTTGTGCTTCACGTTTAGGGGTTAAGACTTGTA

**Chromosome 5B**

>TP8562_query

CAGCACATATCGATTATAGAGATCAAGTATGGATCAAATAACATCACATGGACATTCACTTCCT

>TP8562_hit

CAGCACATATCGATTATAGAGATTAAGTATGGATCAAATAACATCACATGGACATTCACTTCCT

>TP47547_query

CTGCAAATGGTTGTTCTTGGCACATTCATGACATATCTACACCAGATGCAAAATCATATAATAT

>TP47547_hit

CTGCAAATGGTTGTTCTTGGCACATTCATGACATTTCTACACCAGATGCAAAATCATATAATAT

>TP47971_query

CTGCAACAAGCATCCTCTCCAAGAAATGGAACCAACTATGGCTCTCAGTTCTCACTCTTGATTT

>TP47971_hit

CTGCAACAAGCATCCTCTCCAAGAAATGGAACCAACTATGGCTCTCCGTTCTCACTCTTGATTT

>TP50569_query

CTGCAATAAATAAATCTTTCAGAACCTTTAATTAAAGGTTCACACTTACAGTTCACACCTCCTT

>TP50569_hit

CTGCAATAAATAAATCTTTCAGAACCTTTAATTAAAGGTTCACACTTACAGTTCACACCTTCT

>TP52871_query

CTGCACAAGTTGGTCAAGGTAGACATGGAAAAGGCTCCGGATTCTCCTTATCGAGTGTTAAAAC

>TP52871_hit

CTGCACAAGTTGGTCAAGGTAGACATGGAAAAGGCTCTGGATTCTCCTTATCGAGTGTTAAAAC

>TP56384_query

CTGCAGACTGTGAAGCTGAGAGAATTCGATCAACATACAATTTCAAGGCGGAAGACATTTCAAA

>TP56384_hit

CTGCAGACTGTGAAGCTGAGAGAATTCGATCGACATACAATTTCAAGGCGGAAGACATTTCAAA

>TP61799_query

CTGCATGTAATGTGCTAAAGCGACATAAATACATTTTGAGACAGGAACGAGTCTTAACTCTTAA

>TP61799_hit

CTGCATGTAATGTGCTAAAGCGACATAAGTACATTTTGAGACAGGAACGAGTCTTAACTCTTAA

>TP81049_query

CTGCTCGTATTCAAAGTGAATGACGAAACATATTAATGCTATCCCCTCTCTTTTTTTGACCAAA

>TP81049_hit

CTGCTCGTATTCAAAGTGAATGACGAAACATATTAATGCTATCCTCTCTCTTTTTTTGACCAAA

>TP80460_query

CTGCTCCGATTGTACGATTTTGACCCAGATAGTGAAGGGAAATCTCTCGTATTTTAAATACGAC

>TP80460_hit

CTGCTCCGATTGTACGATTTTGACCCAGATAGTGAAGGGAAATCTCTCGTATTTTAACTACGAC

**Chromosome 7A**

>TP13294_query

CAGCAGTACAACAGAAAAAGTTTGGTCTCAGTTGCGGCAACAATTTAGACTTGTGTAGTATAAT

>TP13294_hit

CAGCAGTACAACAGAAAAAGTTTGGTCTCAGTTGTGGCAACAATTTAGACTTGTGTAGTATAAT

>TP32322_query

CAGCTAAAATTGACATTTTTCATCCTTGGGAACGAGATTGAGGCCAATGCTCCTCAATTCATGT

>TP32322_hit

CAGCTAAAATTGACATTTTTCATCCTTGGGAACGAGATTGAGGCCAATTCTCCTCAATTCATGT

**Chromosome 8A**

>TP9964_query

CAGCACGCAATCGGTTGGTCCTTTGTGAATAATATATCTTTGTGGCAAGAGGTGCAGGAAAATC

>TP9964_hit

CAGCACGCAATCGGTTGGTCCTTTGTGAATAATATATCTTTGTGGCAAGAGGTGCAGTAAAATC

>TP34845_query

CAGCTATCCCGGGTTCAGCAACAAATGTAAGAGCTTACCGTCTTTGAAAATTATTATTGAACTG

>TP34845_hit

CAGCTATCCCGGGTTCAGTAACAAATGTAAGAGCTTACCGTCTTTGAAAATTATTATTGAACTG

>TP8423_query

CAGCACAGCCACCACGTCCATCAAGAGCAGAGCAGGTTTCTTCCGTCGCTGGACCTACAGAATC

>TP8423_hit

CAGCACAGCCACCACGTCCATCAAGAGCAGAGCAGGTTTCTTCCGTCGCTGGACCTGCAGAATC

**Chromosome 8D**

>TP2543_query

CAGCAACAACCACTTGTTTGGCCTCTATCTCATCAGCTATGACCACTGCATTGAACTGCTCTCG

>TP2543_hit

CAGCAACAACCACTTGTTTGGCCTCTATCTCATCAGCTATGACCACTGCATTGAACTGCTCTTG

>TP88682_query

CTGCTTTCAGATTGAACATTAGAACCAAACGAAGAAAGTCTTAATAGTGTTCCCTGTGTTTTAG

>TP88682_hit

CTGCTTTCGGATTGAACATTAGAACCAAACGAAGAAAGTCTTAATAGTGTTCCCTGTGTTTTAG
