# Supplementary material for: Mapping freezing tolerance QTL in alfalfa: based on indoor phenotyping
Source: BMC Plant Biol. 2021 Sep 6;21:403. doi: 10.1186/s12870-021-03182-4 (PMC8419964; doi:10.1186/s12870-021-03182-4)
Supplement: Supplementary file 2 — Additional file 2: Supplementary File S2. Fasta sequence of M. truncatula cold tolerance related gene dehydration-responsive element-binding protein 1C (also known as CBF5) obtained from the National Center for Biotechnology Information (NCBI). [file 12870_2021_3182_MOESM2_ESM.docx]

**Supplementary File S2.** *M*. *truncatula* cold tolerance related gene dehydration-responsive element-binding protein 1C (also known as *CBF5*) fasta sequence obtained from National Center for Biotechnology Information (NCBI)

>NC_053047.1:27449437-27451092 Medicago truncatula cultivar Jemalong A17 chromosome 6, MtrunA17r5.0-ANR, whole genome shotgun sequence

AACGTGGCTTGTTTTGAGCCAAATGTTCCTCTTTCATTGTAACTGTCGCTACACACAAGCACACTTTTTC

TGTCCCTACTCTCTCTTTTCTCATCTGTTTCCCTTTCTCTTTCCAAACATTTCTTGACCAATCACAAAAC

CTCCAACTTGGCAATTAGCTCGCTCACACAAAACACACACACTCACAAACACTTGCCTGTTACCTTATTC

TTTCCTTCAAACACAACCATGCTTCCCTATATATATAACACACCTTACTCTTCATTCTCTTAAAACAAAC

TCTCTCCAATTCCCACTTTACCTCTCAAAACCATTTTCCACTCTATCCAACACATACATATGATTAGTAC

TAACAACTCTTCCTATTCACACTCCATTTCCTCAAAAGATTTTTCTCCCTTCGACGCATCATCACCGGGT

TCTGAGGTGCGGTTAGCATCAAGTAACCCGAAGAAGCGAGCAGGGAGGAAGATATTTAAGGAAACTCGCC

ACCCGGTCTATAGAGGTGTGAGGAAGAGGAACTTAAATAAATGGGTTTGTGAAATGAGGGAGCCGAACAC

AAAGAATAGGATTTGGCTGGGAACTTTTCCAACACCGGAGATGGCTGCCCGAGCCCACGATGTTGCTGCA

ATGGCATTGAGGGGCCGCTATGCCTGTCTCAATTTCTCAGACTCGGTGTGGCGCCTTCCTATTCCAGCAA

CTTCCGCAATAAAAGATATTCAAAAGGCAGCTACAAAAGCGGCCGAAGCTTTTAGACCAGACAATACTTT

AATGACTAACAATATTGACACTATCGTAGATGTCGTCGCCACAAAGGAGCTGAATATGTTTTGTGTGGAA

GTTGATGAACAGGAGGAAATGTTGAATATGCCAGAGTTGTGGAGGAATATGGCGTTGATGTCACCTACAC

ATAGCTTTGAGTATCATGAGTATGACGATATTCATGTACAAGATTTTCAAGATGATGAGGTATCACTATG

GAACTTTTAAATTTAATGTCTTTTGTTTACTTTTCTTTTTGGATTGTAGGATAGGAAATGTCTATACTAC

AACCTTTTTAATTGGTAGCATAAATACTGCATATACATAGGTTAAAATTAAAAAGGAATTTCTTTTTATA

GCTTTTTATGTGAATATTAAATCACTAATCGAGCATATAGTGCATCTCATGACTTTTATTATATATGAAA

AAAGAATATAATTTTGGAATTTTCCAAATATAACCTATTCAGTTGGAATTTCAGCTTTTCACATAGAACA

TTTATCTACTGCTATCCTTGTTTTGGTTATACTAAGTTGTAGGAGGTAAAATAGTGTATCTTGTCTTAAT

TTTCTCTTCTCTTTTGTAGGACTTAAAAAAAAAGTCTGTTACCACTACCTGGACTGTGACTGCAACTGGG

GTGCATTCGCCGCATTTCACCATAATGTACAGGATCGTGATTGTTGCCGTGACTGCGAACCCCGCTTTTA

TATTTTCTTTGTTTTCTTCCTGGCAAAGAAAGGTAGAAAAGAAGAAAGAATAAAACTTGAATGGTCATAT

TCTGAATTTACTACCTAAGCAGAAGCCAGTGATGAATACACTCGGTAATTTCTTCATGTGTTTGATCCAG

AAGGGTATGTTTATAGCTGTCTGAATGGTCATTGCCCCTTTTCCAA
